# The impact of COVID-19 restrictions on perceived health and wellbeing of adult Australian sport and physical activity participants

**DOI:** 10.1186/s12889-022-13195-9

**Published:** 2022-04-28

**Authors:** R. Eime, J. Harvey, M. Charity, S. Elliott, M. Drummond, A. Pankowiak, H. Westerbeek

**Affiliations:** 1 School of Science, Psychology and Sport, Federation University, Ballarat, Australia; 2 Institute for Health and Sport, Victoria University, Footscray, Australia; 3 College of Education, Psychology and Social Work, Flinders University, Adelaide, Australia

**Keywords:** Community sport, Health, Adults

## Abstract

Individuals’ access to sport and physical activity has been hampered due to COVID-19 lockdown restrictions. In Australia participation in community sport was cancelled during lockdowns. There is limited research on the impact of sport participation restrictions on the health and wellbeing of adults.

**Aim**

The aim of this study was to investigate the perceived health and wellbeing of a sample of predominantly active Australian adults, both during COVID-19 and in comparison with one year earlier (pre COVID-19).

**Methods**

A survey was conducted during the first COVID-19 restrictions and lockdowns in Australia in May–June 2020. It was distributed by national and state sporting organisations and through researchers’ social media accounts. This particular paper focuses on adults aged 18–59 years. The survey collected information on participant demographics, the sport and physical activity patterns pre- COVID-19, and health and wellbeing outcomes during COVID-19 lockdown and compared to one year earlier. The health measures were cross-tabulated against the demographic and sport and physical activity variables, and group profiles compared with chi-square tests. Scales were derived from three wellbeing questions, and group differences were analysed by t-tests and F-tests.

**Results**

The survey sample included 1279 men and 868 women aged 18–59 years. Most (67%) resided in metropolitan cities. The great majority (83%) were sport participants. During COVID-19 lockdown men were significantly more likely than women to report worse or much worse general (*p* = 0.014), physical (*p* = 0.015) and mental health (*p* = 0.038) and lower life satisfaction (*p* = 0.016). The inactive adults were significantly more likely to report poorer general health (*p* = 0.001) and physical health (*p* = 0.001) compared to active adults. The younger age cohort (18–29 years) were significantly more likely to report poorer general wellbeing (*p* < 0.001), and lower life satisfaction (*p* < 0.001) compared to the older age groups.

**Conclusion**

It seems that the absence of playing competitive sport and training with friends, teams and within clubs has severely impacted males and younger adults in particular. Sports clubs provide an important setting for individuals’ health and wellbeing which is why clubs require the capacity to deliver sport and individuals may need to regain the motivation to return.

## Introduction

Australia had its first reported COVID-19 case in January 2020, and on March 11^th^ the World Health Organisation declared COVID-19 a pandemic, and following this, all Australian borders were closed on March 25^th^ [1]. From March to October 2020 there was widespread cancellation of elite and community sport in Australia. In May 2020 ‘return to sport guidelines’ were developed by National and State Governments, and by mid-October 2020 restrictions were significantly eased in the Australian state most impacted thus far, Victoria [1]. In October 2021, participation in competitive sport was still not allowed with a full return to competitive sport once vaccination rates have increased [2]. In Australia, the state of Victoria has been more impacted by restrictions and lockdowns than any other state, in terms of both length and intensity of restrictions, and the state capital Melbourne has become the world’s most locked-down city [3].

Restrictions on physical movement and limitations on social connections have been shown to significantly affect physical and mental wellbeing [4–6]. A systematic review investigated the impact of COVID-19 on mental health in the general population across 8 countries and reported relatively high rates of anxiety, depression, post-traumatic stress disorder, psychological distress [4]. Other studies have reported that social distancing, self-isolation, and lockdown are among the major contributing factors towards high feelings of sadness, fear, frustration, feelings of helplessness, loneliness, and nervousness [5]. A multi country study of over 1,000 study participants reported that COVID-19 home confinement had a negative effect on both mental health as well as on mood and feelings compared to pre-COVID-19 restrictions [6].

The impacts can also be exacerbated in many cases due to limits on participation in sport and other leisure activities [7–10], which can also be dependent on the type of sport or physical activity that people were involved in pre-COVID-19 as well as during-COVID-19. The restrictions across the globe included limits on time spent outdoors and resulted in reductions in leisure-time sport and physical activity [8]. However, while sport was cancelled, the government in Australia encouraged people to be active, and being active was one of a very few reasons people could leave their homes [2]. The World Health Organisation provided recommendations on how individuals could still meet the recommended physical activity guidelines of 150 min/wk of moderate to vigorous physical activity (MVPA) or 75 min/wk of vigorous physical activity (VPA) for adults, even at home with no special exercise equipment and with limited space [11].

Globally, individuals are more likely to be active in non-sporting activities like walking, running, cycling and swimming rather than participation in sport [12]. Further, children and youth are far more likely to participate in sport than adults and older adults [12–14]. Internationally popular sports for adults include soccer, basketball, golf and tennis [12]. For adults, in Australia popular sport activities include swimming, soccer, tennis, basketball, golf and netball [15, 16].

In terms of restrictions on activity, when easing the restrictions out of total lockdown, some constraints with regard to participation in sport were lifted for children and youth but not for adults [17]. For example, in Regional Victoria children could return to playing sport outdoors, including team-based competitive contact sport, but adults could only train. Indoor sports and physical activities remained prohibited at this time, so that sports such as football and tennis resumed for children and youth, but not basketball or other gym-based activities [17]. COVID-19 restrictions will continue to impact children and youth differently compared to adults and older adults.

The impact on physical, social and mental health, as well as the economic impact, of COVID-19 varies across individuals and families, according to demographic factors such as gender, age and residential region (metropolitan versus regional and rural) [5, 18, 19].

Regarding gender, there is evidence that women may suffer greater psychological strain and stress and anxiety and depression compared to men. This can be attributed to a lockdown situation where all family members are at home and where women, more than men, had to juggle home duties with work commitments, which can be mentally and physically demanding [5]. This is consistent with another study reporting that women had higher rates of mental health problems than men [18]. This can relate to physical activity and work status. For example, sport participants are much more likely to be male than female, and this is consistent internationally [14, 20, 21]. However, in terms of impact of COVID-19 on gender, an international study investigated COVID-19 impacts on gender gaps in economic outcomes, and reported that women were more likely to permanently lose their job than men [22]. This study was conducted across 6 countries including China, South Korea, Japan, Italy, the United Kingdom and four of the largest states in the United States [22]. Further, there are also reports that the closure of schools across many nations might have a differential impact on women as they provide most of the informal care within families and as a consequence may have to limit their work [23]. Further, women are less likely to have decision making power in context of pandemic related matters and therefore their opinions and needs are less considered [23].

There are also age-related trends in the impact of COVID-19 on mental health with an Austrian study of adults reporting that mental health problems were higher in adults aged under 35 years, as well as among people who were out of work or on low incomes [18]. The requirement to socially distance during lockdown and other restrictions on the number of people congregating outside the home or the ability to visit people in their home has placed insurmountable pressure on social relationships and therefore social health and wellbeing. This in turn also affects mental health. For example, economic hardship can impact the quality and stability of couples’ relationships [24]. Further, in a UK study among younger adults, those separated or divorced were more likely to suffer poorer mental health [25].

In Australia participation in community sport is much more popular in regional and rural areas compared to metropolitan [14]. More specifically, the metropolitan growth areas have lower than average participation compared to longer established suburbs [26]. This can be explained by many factors including the (important) social nature of community sport in regional and rural areas, the availability of traditional club-based community sport versus other leisure-time activities and socio-economic status, as well as population density [26, 27].

In terms of impact of COVID-19 by region, to date there have been considerable differences across states and also within states. Regional and rural areas had lower numbers of COVID-19 cases and were less impacted by social restrictions and lockdowns than metropolitan areas, and most specifically Melbourne in Victoria [28]. Therefore, the ability of people to be active and return to activities including sport and indoor leisure varied considerably across regions and across timepoints [2]. There was also a significant seasonal impact in Australia in 2020 with summer sports like cricket and tennis largely unaffected. However, most winter sports like Australian football, soccer and netball, which generally run April-August had to cancel their competitions for almost the whole season.

Internationally, there were different impacts of COVID-19 between regions, which can be attributed to population density but also demographic factors such as age. In Australia, pre-COVID-19 there were already differences in health status according to residential location. For example, a lack of health care services in regional and rural areas, poor internet leading to limited telehealth, and a lack of social capital and availability of social services makes rural communities particularly vulnerable compared to metropolitan cities [19]. However, metropolitan populations were at risk due to community spread from higher population density [19].

There is limited research on adults in regard to their community level sport involvement. Most research has targeted the young and the elite sport participants, the majority of which tend to focus on general physical activity and not specifically on sport. In this paper we seek to determine the association between perceived health and wellbeing of adults and the impact of COVID-19 related restrictions on different types and settings of participation in sport and physical activity, together with different genders, age groups and regions.

## Methods

This study is part of a broader program of research in Australia, which involves the longitudinal measurement of sport and physical activity participation and the physical, mental and social health and wellbeing outcomes of this participation. This study was conducted via two waves of online surveying (Qualtrics) during the COVID-19 period (2020 and 2021), the first of which also included participation and health data that related to the pre-COVID-19 baseline in 2019. Ethics approval was obtained by Flinders University (project number 8654) and Victoria University (project number HRE20-049) human research ethics committees.

The present study is based on data collected in the first wave using an online survey of sport participants conducted during May and June 2020. Recruitment to the survey was primarily facilitated by sports including Australian football, bowls, cricket, golf, tennis and football. The present study is one of three age-based studies, each focusing on a different stage of the lifespan. The other studies are focused on adolescence (13–17 years) and older adulthood (60 + years). The present study is focused on early and middle adulthood. The target population for this paper was adults aged 18–59 years at the time of the survey who were registered in the 2019 and/or 2020 playing seasons to participate in one or more sports. Separate analysis and papers are being developed for youth and older adults. The sports organisations that sent out the invitation to the survey to their registered participants represent major sports in Victoria and Australia [16, 29]. The research team has extensive research experience in working with these sports at national, state and local levels [10, 30–35].

In order to broaden the scope of the survey sample to include people who participate in recreational physical activity only in settings other than sports clubs, and potentially also people who do not participate in any recreational physical activity, the primary recruitment strategy was supplemented by the use of snowball sampling, through social media pages of sports organisations and research-oriented social media pages (e.g. research team social media pages, university social media pages and websites).

The first wave, or baseline, of the longitudinal survey included, among others, questions about:

- Demographic characteristics – gender, date of birth, and residential postcode
- Types of sports and other recreational physical activities participated in
- Settings in which the participation occurred – sports clubs and other less structured informal settings
- Modes of participation – team and individual modes of activity
- Self-assessed general health, physical health and mental health.
- Measures of wellbeing – general wellbeing, resilience and life satisfaction.

Date of birth was used to determine age in years at the time the survey was completed. Age was then recoded into three age cohorts (18–29 years, 30–49 years, and 50–59 years), broadly aligning with ‘young and free’, ‘homebuilding and child rearing’, and ‘empty nesting’. Residential postcode concordances [36] were used to assign each postcode to one of two broad geographical zones or regions: Metropolitan, comprising the capital cities of the Australian states; and Non-metropolitan, comprising regional cities, towns and rural areas.

Regarding sport and physical activity, two separate sections of the survey dealt respectively with two ‘sport and physical activity settings’: organised club sport involving membership and registration (designated ‘club’), and less structured sport and recreational physical activity (designated ‘informal’). In each section, a list of the most common activities was presented – 16 for club sports and 26 for informal (which also included 12 of the 16 club sports). Respondents indicated the activities in which they participated, with provision for adding other activities that were not listed. Based on these responses, a combined list of 88 activities was established. Further, each of the 88 activities was classified as either ‘team’ or ‘individual’, which we refer to as ‘sport and physical activity modes’. Each respondent was then assigned a single overall category for each of settings (club only, club and informal, informal only, and inactive) and modes (team only, team and individual, individual only, inactive).

Six survey items were devoted to self-assessed health – three pertaining to the time of the survey (during COVID-19 lockdown) and three comparing current health to health 12 months prior to the survey (before COVID-19). The general health item was a 5-point Likert scale item (poor, fair, good, very good, excellent) derived from the Short-form Health Survey (SF-36) instrument [37]. The same format was used for the assessment of physical health and mental health. The three comparative items used a 5-point Likert scale (much worse, somewhat worse, about the same, somewhat better, much better).

General wellbeing was assessed using a scale derived by averaging the responses to a battery of 14 items regarding frequency of positive and negative feelings, derived from [38]. Each item was scored on a 5-point scale (all of the time, most, some, a little, none), with reverse coding of the negative items, so that higher average scores represented greater wellbeing.

Resilience was similarly assessed using a scale derived by averaging the responses to a battery of 4 items derived from [39]. Each item consisted of a statement about the respondent, with responses on a 5-point scale (strongly agree, agree, neutral or unsure, disagree, strongly disagree).

Life satisfaction was assessed using a direct question {Women's Health Australia, 2008 #2448} with the response on a 10-point scale from 1 (least satisfied) to 10 (most satisfied).

### Statistical analysis

Data analysis was conducted using SPSS Version 25. For the purpose of tabulation and statistical analysis of responses, each of the six 5-category health items were recoded into three categories (poor/fair, good, very good/excellent; and much/somewhat worse, about the same and somewhat/much better). Similarly, the variable ‘settings of sport and physical activity’ was recoded from four categories (club only, club and informal, informal only, inactive) to three (club including club and informal, informal only, inactive) and the variable ‘modes of sport and physical activity’ was recoded from four categories (team only, team and individual, individual only, inactive) to three (team including team and individual, individual only, inactive).

The six recoded health items were each cross-tabulated against five respondent characteristics: gender, age cohort, region, settings of sport and physical activity, and modes of sport and physical activity. Chi-square tests of independence were conducted to identify differences in the health profiles of the groups defined by each of the characteristics.

For the measures of general wellbeing, resilience and life satisfaction, mean values for the groups defined by each of the five characteristics were tabulated, and group differences were analysed using independent samples t-tests (for two groups) and F-tests (for three groups).

## Results

The survey was completed by 2,146 adults aged 18–59 years. Table 1 shows profiles of gender, age groups and region of residence, for the survey sample and for the corresponding age cohort of the Australian population. Men were over-represented and women under-represented in the survey, which is consistent with the known higher rate of sports participation by men, and the fact that the distribution of survey invitations was facilitated predominantly by the organisations of male-dominated sports. The younger and middle-aged adults (18–49 years), were also under-represented, which is consistent with the known drop in sports participation during adolescence, with lower participation continuing through the years of higher education, the pursuit of careers and the establishment and raising of families. Participation rates rose again during the “empty nest” ages of 50–59 years. The two types of region were close to proportionately represented in the survey sample, with a slight over-representation of non-metropolitan areas which may reflect the more central role of traditional forms of sport in these areas.

**Table 1:** Demographic profiles of survey sample, compared to Australian population aged 18–59 years

	Survey Sample	Australian Population^a^
	%	%
Gender		
Male	59.6	49.7
Female	40.4	50.3
Age (years)		
18–29	25.0	29.3
30–49	44.9	49.0
50–59	30.1	21.7
Region		
Metropolitan	67.0	70.5
Non-metropolitan	33.0	29.5

^a^Source: Australian Bureau of Statistics. Regional population by age and sex, 2020 [40]

Consistently with the methods of recruitment, the great majority of survey repondents were participants in club sport (*n* = 1779, 83%). Fewer played only informally (*n* = 356, 17%), and very few were inactive (*n* = 20, < 1%). The majority played team sport (*n* = 1455, 68%), and fewer participated in only individual activities (*n* = 680, 32%).

### Reported health status during COVID-19 restrictions/lockdowns

There were significant gender differences in reported general health (*p* < 0.001), physical health (*p* = 0.006) and mental health (*p* = 0.23) during COVID-19 restrictions/lockdowns. Men were more likely than women to report poor/fair general health (22%, 16%), poor/fair physical health (26%, 21%) and poor/fair mental health (34%, 28%), whereas women were more likely than men to report very good or excellent general health (51%, 42%), physical health (43%, 37%) and mental health (35%, 33%) (Table 2).

**Table 2:** Self-assessment of current health: by respondent characteristics

Health assessments	Characteristics						*p*-value^a^
	Gender^b^						
	Male		Female				
	N	%	N	%			
General health							< .001
Poor or fair	279	21.8	142	16.4			
Good	463	36.2	283	32.6			
Very good or excellent	536	41.9	443	51.0			
Total	1278	100.0	868	100.0			
Physical health							.006
Poor or fair	332	26.0	182	21.0			
Good	477	37.3	314	36.3			
Very good or excellent	469	36.7	369	42.7			
Total	1278	100.0	865	100.0			
Mental health							.023
Poor or fair	429	33.6	243	28.0			
Good	431	33.8	319	36.8			
Very good or excellent	415	32.5	305	35.2			
Total	1275	100.0	867	100.0			
	Age (years)						
	18–29		30–49		50–59		
	N	%	N	%	N	%	
General health							
Poor or fair	123	22.8	201	20.8	100	15.5	.017
Good	183	34.0	335	34.6	228	35.2	
Very good or excellent	233	43.2	432	44.6	319	49.3	
Total	539	100.0	968	100.0	647	100.0	
Physical health							
Poor or fair	131	24.4	250	25.8	136	21.1	.115
Good	184	34.3	361	37.3	246	38.1	
Very good or excellent	222	41.3	357	36.9	264	40.9	
Total	537	100.0	968	100.0	646	100.0	
Mental health							
Poor or fair	214	39.7	304	31.5	157	24.3	< .001
Good	177	32.8	355	36.8	220	34.1	
Very good or excellent	148	27.5	306	31.7	269	41.6	
Total	539	100.0	965	100.0	646	100.0	
	Region						
	Metropolitan		Non-metropolitan				
	N	%	N	%			
General health							.432
Poor or fair	291	20.3	129	18.3			
Good	486	33.8	255	36.1			
Very good or excellent	660	45.9	322	45.6			
Total	1437	100.0	706	100.0			
Physical health							
Poor or fair	346	24.1	166	23.6			.066
Good	505	35.2	282	40.1			
Very good or excellent	585	40.7	256	36.4			
Total	1436	100.0	704	100.0			
Mental health							
Poor or fair	465	32.4	207	29.5			.090
Good	480	33.4	268	38.2			
Very good or excellent	492	34.2	227	32.3			
Total	1437	100.0	702	100.0			
	Sport and physical activity settings						
	Club^c^		Informal only^d^		Inactive		
	N	%	N	%	N	%	
General health							.143
Poor or fair	356	19.9	61	17.7	7	35.0	
Good	616	34.4	121	35.1	9	45.0	
Very good or excellent	817	45.7	163	47.2	4	20.0	
Total	1789	100.0	345	100.0	20	100.0	
Physical health							.023
Poor or fair	430	24.1	77	22.3	10	50.0	
Good	660	37.0	123	35.7	8	40.0	
Very good or excellent	696	39.0	145	42.0	2	10.0	
Total	1786	100.0	345	100.0	20	100.0	
Mental health							
Poor or fair	571	32.0	96	27.9	8	40.0	.286
Good	615	34.4	133	38.7	4	20.0	
Very good or excellent	600	33.6	115	33.4	8	40.0	
Total	1786	100.0	344	100.0	20	100.0	
	Sport and physical activity modes						
	Team^e^		Individual only^f^		Inactive		
	N	%	N	%	N	%	
General health							.096
Poor or fair	291	20.0	126	18.5	7	35.0	
Good	510	35.1	227	33.4	9	45.0	
Very good or excellent	653	44.9	327	48.1	4	20.0	
Total	1454	100.0	680	100.0	20	100.0	
Physical health							
Poor or fair	344	23.7	163	24.0	10	50.0	.034
Good	539	37.1	244	35.9	8	40.0	
Very good or excellent	568	39.1	273	40.1	2	10.0	
Total	1451	100.0	680	100.0	20	100.0	
Mental health							
Poor or fair	452	31.2	215	31.7	8	40.0	.646
Good	505	34.8	243	35.8	4	20.0	
Very good or excellent	494	34.0	221	32.5	8	40.0	
Total	1451	100.0	679	100.0	20	100.0	

^a^Chi-square test of independence

^b^Seven respondents who reported their gender as’Other’ were excluded from the gender breakdowns because the small sample size provided inadequate statistical power to enable reliable conclusions to be drawn about this population

^c^Those who participated in club sports, including those who also participated in informal sport or other recreational physical activities

^d^Those who participated in informal sport or other recreational physical activities, but not in club sports

^e^Those who participated in team sports or activities, including those who also participated in individual sports or activities

^f^Those who participated in individual sports or physical activities, but not in team sports or activities

When broken down by age categories there were significant differences for general health (*p* = 0.017) and mental health (*p* < 0.001). In both cases, the younger cohort (18–29 years) were more likely to report poor/fair general health (23%) and poor/fair mental health (40%) compared to older adults, with those aged 50–59 years being more likely to report very good/excellent general health (50%) and very good/excellent mental health (42%) (Table 2).

There were no significant regional differences in the three health measures (Table 2).

With regard to the level physical activity, inactive adults were significantly more likely to report poor/fair physical health compared to active adults in either informal activities or club-based sport (*p* = 0.02). Further, inactive adults were significantly more likely to report poor/fair physical health compared to those active within team or individual activities (*p* = 0.034).

### Reported changes in health status: before and during COVID-19 restrictions/lockdowns

Table 3 summarises the results of self-assessed health during COVID-19 lockdown compared to a year before (and pre-COVID-19). Men were more likely than women to report worse/much worse general health (33%, 30%; *p* = 0.014), worse/much worse physical health (38%, 33%; *p* = 0.15) and worse/much worse mental health (42%, 37%; *p* = 0.038).

**Table 3:** Self-assessment of current health compared to one year ago: by respondent characteristics

Health assessments	Characteristics						*p*-value^1^
	Gender^2^						
	Male		Female				
	N	%	N	%			
General health							
Worse or much worse	425	33.2	242	27.9			.014
About the same	582	45.5	407	46.9			
Better or much better	272	21.3	219	25.2			
Total	1279	100.0	868	100.0			
Physical health							
Worse or much worse	478	37.5	285	32.9			.015
About the same	517	40.5	348	40.1			
Better or much better	281	22.0	234	27.0			
Total	1276	100.0	867	100.0			
Mental health							
Worse or much worse	532	41.6	320	36.9			.038
About the same	532	41.6	372	42.9			
Better or much better	215	16.8	176	20.3			
Total	1279	100.0	868	100.0			
	Age (years)						
	18–29		30–49		50–59		
	N	%	N	%	N	%	
General health							
Worse or much worse	205	38.0	313	32.3	153	23.6	< .001
About the same	191	35.4	437	45.1	362	56.0	
Better or much better	143	26.5	219	22.6	132	20.4	
Total	539	100.0	969	100.0	647	100.0	
Physical health							
Worse or much worse	219	40.7	365	37.7	183	28.3	< .001
About the same	163	30.3	377	39.0	326	50.5	
Better or much better	156	29.0	225	23.3	137	21.2	
Total	538	100.0	967	100.0	646	100.0	
Mental health							
Worse or much worse	236	43.8	399	41.2	220	34.0	< .001
About the same	173	32.1	395	40.8	339	52.4	
Better or much better	130	24.1	175	18.1	88	13.6	
Total	539	100.0	969	100.0	647	100.0	
	Region						
	Metropolitan		Non-metropolitan				
	N	%	N	%			
General health							
Worse or much worse	451	31.4	216	30.6			.315
About the same	647	45.0	340	48.2			
Better or much better	340	23.6	150	21.2			
Total	1438	100.0	706	100.0			
Physical health							
Worse or much worse	519	36.2	244	34.6			.312
About the same	562	39.2	300	42.6			
Better or much better	354	24.7	161	22.8			
Total	1435	100.0	705	100.0			
							.057
Mental health							
Worse or much worse	590	41.0	262	37.1			
About the same	580	40.3	323	45.8			
Better or much better	268	18.6	121	17.1			
Total	1438	100.0	706	100.0			
	Sport and physical activity settings						
	Club^3^		Informal only^4^		Inactive		
	N	%	N	%	N	%	
General health							.001
Worse or much worse	583	32.6	79	22.9	9	45.0	
About the same	818	45.7	165	47.8	7	35.0	
Better or much better	389	21.7	101	29.3	4	20.0	
Total	1790	100.0	345	100.0	20	100.0	
Physical health							.002
Worse or much worse	665	37.2	92	26.7	10	50.0	
About the same	710	39.8	150	43.5	6	30.0	
Better or much better	411	23.0	103	29.9	4	20.0	
Total	1786	100.0	345	100.0	20	100.0	
Mental health							< .001
Worse or much worse	742	41.5	107	31.0	6	30.0	
About the same	746	41.7	154	44.6	7	35.0	
Better or much better	302	16.9	84	24.3	7	35.0	
Total	1790	100.0	345	100.0	20	100.0	
	Sport and physical activity modes						
	Team^5^		Individual only^6^		Inactive		
	N	%	N	%	N	%	
General health							
Worse or much worse	465	32.0	197	29.0	9	45.0	.423
About the same	658	45.2	325	47.8	7	35.0	
Better or much better	332	22.8	158	23.2	4	20.0	
Total	1455	100.0	680	100.0	20	100.0	
Physical health							
Worse or much worse	529	36.4	228	33.6	10	50.0	.335
About the same	569	39.2	291	42.9	6	30.0	
Better or much better	354	24.4	160	23.6	4	20.0	
Total	1452	100.0	679	100.0	20	100.0	
Mental health							
Worse or much worse	576	39.6	273	40.1	6	30.0	.237
About the same	625	43.0	275	40.4	7	35.0	
Better or much better	254	17.5	132	19.4	7	35.0	
Total	1455	100.0	680	100.0	20	100.0	

^1^Chi-square test of independence

^2^Seven respondents who reported their gender as’Other’ were excluded from the gender breakdowns because the small sample size provided inadequate statistical power to enable reliable conclusions to be drawn about this population

^3^Those who participated in club sports, including those who also participated in informal sport or other recreational physical activities

^4^Those who participated in informal sport or other recreational physical activities, but not in club sports

^5^Those who participated in team sports or activities, including those who also participated in individual sports or activities

^6^Those who participated in individual sports or physical activities, but not in team sports or activities

When broken down by age, the youngest cohort, aged 18–29 years, were significantly more likely to report worse/much worse general health (38%; *p* < 0.001), worse/much physical health (41%; *p* < 0.001) and worse/much mental health (44%; *p* < 0.001) compared to the older cohorts (Table 3).

There were no significant differences in the reported change in health status for those living in metropolitan cities compared to non-metropolitan regions.

The inactive adults were significantly more likely to report worse/much worse general health (*p* = 0.001) and worse/much worse physical health (*p* = 0.001) compared to those active through club or other informal activities. However, the adults who were active through clubs and other informal activities were significantly more likely to report worse/much worse mental health (*p* < 0.001) compared to the inactive adults (Table 3).

### Measures of wellbeing

The results of general wellbeing, resilience and life satisfaction are presented in Table 4. Men reported significantly lower life satisfaction (mean 6.63; *p* = 0.16) than women (mean 6.82). The younger age cohort (18–29 years) reported significantly poorer general wellbeing (*p* < 0.001), and lower life satisfaction (*p* < 0.001) than the older age groups. There were no significant differences by region, activity setting or activity mode.

**Table 4:** Measures of wellbeing^a^: by four respondent characteristics

Measure	Characteristics									*p*-value^b^
	N	Mean	SD	N	Mean	SD	N	Mean	SD	
	Gender^c^									
	Male			Female						
General wellbeing	1219	3.43	0.66	829	3.45	0.66				.601
Resilience	1204	3.69	0.67	823	3.68	0.70				.610
Life satisfaction	1212	6.63	1.80	830	6.82	1.66				.016
	Age (years)									
	18–29			30–49			50–59			
General wellbeing	511	3.28	0.69	929	3.42	0.65	615	3.58	0.64	< .001
Resilience	498	3.62	0.72	917	3.70	0.67	618	3.72	0.67	.063
Life satisfaction	501	6.44	1.78	925	6.72	1.70	623	6.91	1.78	< .001
	Region									
	Metropolitan			Non-metropolitan						
General wellbeing	1364	3.43	0.67	681	3.44	0.66				.664
Resilience	1353	3.71	0.68	670	3.64	0.69				.034
Life satisfaction	1364	6.69	1.76	675	6.75	1.74				.775
	Sport and physical activity settings									
	Club^d^			Informal only^e^			Inactive			
General wellbeing	1700	3.44	0.67	336	3.44	0.62	19	3.20	0.96	.300
Resilience	1684	3.69	0.69	332	3.67	0.67	17	3.56	0.58	.646
Life satisfaction	1698	6.69	1.78	334	6.83	1.57	17	6.47	2.53	.334
	Sport and physical activity modes									
	Team^f^			Individual only^g^			Inactive			
General wellbeing	1381	3.43	0.65	655	3.44	0.68	19	3.20	0.96	.294
Resilience	1360	3.69	0.69	656	3.68	0.69	17	3.56	0.58	.675
Life satisfaction	1373	6.70	1.74	659	6.72	1.77	17	6.47	2.53	.837

^a^General wellbeing: 14 items, scale 1–5. Resilience: 4 items, scale 1–5. Life satisfaction: 1 item, scale 1–10

^b^Two groups: independent samples t-test; three groups: F-test

^c^Seven respondents who reported their gender as’Other’ were excluded from the gender breakdowns because the small sample size provided inadequate statistical power to enable reliable conclusions to be drawn about this population

^d^Those who participated in club sports, including those who also participated in informal sport or other recreational physical activities

^e^Those who participated in informal sport or other recreational physical activities, but not in club sports

^f^Those who participated in team sports or activities, including those who also participated in individual sports or activities

^g^Those who participated in individual sports or physical activities, but not in team sports or activities

## Discussion

This study reports on the perceived health of Australian adults during the first period of COVID-19 restrictions and lockdowns in 2020, and compared those health status measures to pre-COVID status. The study sample was predominantly physically active, and most were active through team-based sports. This bias was due to the nature of the survey distribution through State Sporting Associations which had a higher uptake than the general social media post invitations.

Globally, individuals were impacted by COVID-19 restrictions, including limits on their ability to leave the home and on the amount and types of physical activity and sport that they could undertake. These restrictions differed in different regions and at different times, with restrictions often changing rapidly. However, in Australia at least, governments realised the importance of people being physically active and this remained one of very few legitimate reasons for people to leave their homes [2]. There were public awareness campaigns, including media campaigns, which were similar to previous public health measures. Further, there was widespread availability of many online activities (such as yoga and Pilates). However, the COVID-19 enforced home confinement was unprecedented, and in a first, public health advice included recommendations for maintaining and increasing physical activity while confined at home [6].

Important findings were that the men reported significantly poorer health outcomes (general, physical, and mental) and life satisfaction than the women. Further, the men reported that their health (general, physical and mental) was significantly worse during COVID-19 lockdowns compared to a year previously, than was the case for women. These findings were surprising, especially given that there is consistent evidence that women across many different countries reported poorer health during COVID-19 [22, 23, 41–43]. This international finding can be due to women being more likely to lose jobs than men due to COVID-19 [22] and more likely to be impacted by school closures and more likely to assist with remote learning of children compared to men [23]. In other multi-national studies including adults from the UK, the US, Canada and Australia, women have been impacted greater through caregiving responsibilities and have reported higher distress and anxiety than men [42]. This is consistent with another UK study which reported that women were at risk of worse mental health during COVID-19 compared to men, and that men had a relatively stable trajectory of mental health across the pandemic compared to women [43]. It is not known why the men in this Australian study reported poorer health and that their health was more heavily impacted negatively during COVID-19 than women. The sample in this study consisted of almost exclusively physically active adults, as opposed to other international health studies, where samples were a more general cross section of the population. Why men seemed to be more negatively impacted than women may relate to this fact that the sample consisted of mostly active men, and that their actual levels of physical activity during COVID-19 were more severely affected or interrupted than activity levels of men in studies that included more inactive respondents. A study of twins reported that a perceived decrease in physical activity or exercise was associated with higher stress and anxiety [44], and it is possible that in our predominantly active sporting sample the men were more likely to have higher physical activity levels pre-COVID and be less active during-COVID than the women. This is also in line with a study reporting that individuals who were in a sports club were likely to exercise less during lockdown [45]. The authors stated that the interests and goals of people who exercise in a sports club compared to those in non-organised forms of physical activity differ [45], and perhaps sports club participants were less likely to be active during lockdown.

Younger (active) adults were significantly more likely than the older adults to report poorer health outcomes and worse health during COVID-19 lockdown compared to a year ago. This is consistent with other studies showing that younger adults’ mental health is more impacted than older adults [41, 46, 47]. A UK study measured mental health during COVID-19 lockdown and found that young adults (18–29 years) were more likely to report worse mental health outcomes [47]. Similarly, an Italian study of adults reported that younger age was associated with increased depression, anxiety and high perceived stress [41]. The authors report that these health outcomes were associated with discontinued work [41]. Further, another UK study reported that psychological distress during lockdown was worse for young adults [46]. However, these studies did not go into any further detail as to why this was the case.

There were no significant differences in the health outcomes for those living in regional areas compared to metropolitan cities. In Victoria, Australia where most of the study participants resided, the COVID-19 lockdown restrictions were harsher and longer in metropolitan Melbourne which may have impacted more heavily on individuals’ health. It could therefore be anticipated that rural residents may have reported better health outcomes than their metropolitan counterparts. However, in Australia the pre-COVID health of non-metropolitan individuals was generally poorer than metropolitan individuals, which can be related to large disparities in access to health care and health services, and to geographical isolation [48], and this may have contributed to the results reported in this study. In an American study of working adults aged 18–64 years, those rural residents reported worse health outcomes related to COVID-19 experiences such as physical and mental health than metropolitan residents [49].

When the physical activity type was broken down by setting (club and informal) and mode (team or individual) there were limited differences in health outcomes. Inactive adults were more likely than active adults to report worse general and physical health compared to a year earlier. However, club sport participants were more likely to report a decline in mental health than those active only in informal settings or those who were inactive. On the one hand, compared to inactive adults, those who were active seemed to have a ‘health buffer’ during the COVID-19 restrictions and lockdowns. On the other hand, the mental health of club sport participants was perhaps more specifically impacted by the loss of the psycho-social benefits of club sport [50–52].

### Limitations

This study is based on data from a convenience sample, predominantly of Australian sports participants recruited with the assistance of NSOs and SSOs of four sports, in May and June 2020. The primary sample was supplemented by recruitment through social media, which resulted in an additional smaller sample of participants in only informal sport or other physical activity settings, and an even smaller sample of physically inactive people. Consequently, the sample is subject to both known and unknown sources of bias, and caution must be exercised in generalising the results. Even within the primary club sport sample, the geographical coverage was uneven, depending on the strength of the relationships between the research team and the SSOs in the various states, and the capacities and priorities of different SSOs in the context of the unfolding COVID-19 situation. Nevertheless, on the other side of the ledger, the sample obtained was extremely large, and because respondents provided information about the multiple sports and other physical activities that they engaged in, there was comprehensive representation of the sporting codes and other types of recreational physical activity that are available in Australia.

## Conclusion

In conclusion, this study of mainly active sporting adults indicates that health and wellbeing of men was significantly more impacted than that of women through the absence of participation in sport due to COVID-19 lockdown restrictions. Further, the younger active sporting adults were more likely to report poorer health outcomes during lockdown than the older adults. Participation in team and club-based sport can play an important role not only for physical health but also for social and psychological health and wellbeing [50]. It seems that the absence of playing competitive sport and training with friends, teams and within clubs has severely impacted men and younger adults in particular. Sports clubs provide an important setting for individuals’ health and wellbeing and sport organisations need to focus on ensuring that clubs have the capacity to rebound and that individuals, both volunteers and participants, are given support and encouragement to return. Clubs may need to also consider how they pay particular attention to re-engaging at-risk men who have experienced worse mental health.

## Data Availability

The datasets used and/or analysed during the current study are available from the corresponding author on reasonable request.

## Abbreviation

COVID-19: Coronavirus Disease of 2019
